# A DFT Study of Phosphate Ion Adsorption on Graphene Nanodots: Implications for Sensing

**DOI:** 10.3390/s23125631

**Published:** 2023-06-16

**Authors:** Ivan Shtepliuk

**Affiliations:** Semiconductor Materials Division, Department of Physics, Chemistry and Biology-IFM, Linköping University, S-58183 Linköping, Sweden; ivan.shtepliuk@liu.se

**Keywords:** graphene nanodot, phosphates, adsorption, DFT, TD-DFT, sensors

## Abstract

The optical properties of graphene nanodots (GND) and their interaction with phosphate ions have been investigated to explore their potential for optical sensing applications. The absorption spectra of pristine GND and modified GND systems were analyzed using time-dependent density functional theory (TD-DFT) calculation investigations. The results revealed that the size of adsorbed phosphate ions on GND surfaces correlated with the energy gap of the GND systems, leading to significant modifications in their absorption spectra. The introduction of vacancies and metal dopants in GND systems resulted in variations in the absorption bands and shifts in their wavelengths. Moreover, the absorption spectra of GND systems were further altered upon the adsorption of phosphate ions. These findings provide valuable insights into the optical behavior of GND and highlight their potential for the development of sensitive and selective optical sensors for phosphate detection.

## 1. Introduction

Phosphate is a ubiquitous chemical compound [1,2], found in soil, water, and even the human body. This nutrient is essential for plant growth and is a part of fertilizers [3]. Phosphates also play a pivotal role in several biological processes, including bone growth [4], cellular metabolism [5], and brain dynamics across sleep [6], as well as are commonly used in industrial processes such as metal cleaning and water treatment. Despite the apparent importance and benefits of phosphates, there is a downside: excessive concentrations of phosphates can have negative effects on both the environment (leading to eutrophication [7] and undesirable algal blooms [8]) and human health (causing kidney disease [9] and hypophosphatemia [10]). All the above highlight the need to improve existing methods of detecting phosphates and develop fundamentally new approaches, including the study and design of new sensitive materials. This is extremely important, especially in light of the rationalization of the use of phosphates in agriculture and industry. More excitingly, detecting deviations in phosphate levels in the human body from the norm can provide valuable information about one’s health status [11], thereby promoting early diagnostics and timely treatment.

Numerous methods have been developed for detecting phosphates, including high-performance liquid chromatography (HPLC) [12], fluorescence spectroscopy [13], electrochemistry (potentiometry [14] and amperometry [15]), and enzyme-based biosensing [16]. Unfortunately, many of these approaches are impractical due to the need for specialized costly equipment and trained personnel, careful calibration, and the synthesis of intricate compounds. Despite the variety of methods available, the requirement for careful handling of the reagents and sophisticated instrumentation may restrict these techniques. In this regard, a strategy that focuses on simplifying and streamlining the detection of phosphates would facilitate the widespread implementation of phosphate ion sensors in diverse fields, such as environmental monitoring and medical diagnostics. In contrast to other methods, colorimetry [17] is a relatively simple technique to implement due to its reliance on observable color changes that may be detected without specialized equipment or expertise. Probably the most popular colorimetric method for phosphate detection is the molybdenum blue method [18], which relies on a sequence of chemical reactions between ammonium molybdate and the target phosphate ions to generate a complex that exhibits a characteristic blue coloration. The degree of coloration, indicative of the concentration of phosphate ions, may be quantified using ultraviolet–visible (UV–vis) absorbance spectroscopy [19], with the intensity of the resulting absorbance spectra being directly proportional to the concentration of phosphate ions. Nevertheless, the utilization of ammonium molybdate as a reactant entails both economic and safety concerns, warranting the exploration of cost-effective, eco-friendly alternatives for the detection of phosphate ions in solution.

Graphene nanodots—small fragments or islands of graphene that exhibit quantum confinement effects due to their reduced size and confinement boundaries [20]—are promising candidates for the development of alternative colorimetric methods to the molybdenum blue method due to their excellent biocompatibility [21], low toxicity [22], and light absorption ability/tunability [22,23,24,25,26]. In contrast to graphene’s rapid response exclusively within the terahertz range [27,28,29,30], graphene nanodots possess the remarkable ability to absorb light across a broad spectrum of wavelengths, extending from the ultraviolet (UV) to the near-infrared (NIR) regions. This light absorption capability of GND is contingent upon their specific size, shape, and chemical composition, emphasizing the influence of these factors on their optical properties. These dots have already demonstrated their effectiveness as colorimetric probes in the detection of various substances [31,32,33,34,35,36,37], and thus, their potential for the development of novel colorimetric techniques for phosphate detection is noteworthy. A colorimetric probe (with a limit of detection (LOD) of 9 μU·mL^−1^) based on Au@Ag nanoparticles and GND has been developed for the determination of the activity of alkaline phosphatase (ALP) [38]. Since ALP can catalyze the hydrolysis of phosphate esters, ALP activity can be used as an indirect measurement of phosphate ions in a sample [39]. Chen et al. developed a novel method for the detection of phosphate ions using single-layered GND with aluminum ions (Al^3+^) [40]. This on-off fluorescent probe demonstrated a LOD of 0.1 μM, with the emission intensity decreasing in the presence of PO_4_^3−^ due to the strong interaction between the phosphate ion and Al^3+^. In another investigation [41], an off–on sensor for phosphate ion detection (with LOD of 0.1 μM) was designed using a combination of GND and europium ions (Eu^3+^). The detection principle was based on the observation that the oxygen-donor atoms in phosphate ions exhibit a greater affinity towards Eu^3+^ ions than the carboxylate groups on the surface of GND. Consequently, the Eu^3+^ ions tend to preferentially interact with the phosphate ions instead of the GND, leading to a restoration of the optical signal of the GND.

The motivation behind this research arises from the necessity to enhance current phosphate detection methods and investigate innovative approaches in light of the environmental and health implications associated with phosphate concentrations. The objective of this study is to establish a theoretical basis for the design and optimization of phosphate ion sensors with specific performance characteristics. Although research on using GND for phosphate detection is underway [39,40,41], it is still in its infancy. More fundamental theoretical research needs to be conducted for a deeper understanding of the physical properties of GND-phosphate complexes to enable more effective utilization of these complexes in sensing applications. Indeed, such properties as phosphate adsorption energy, charge transfer direction/magnitude and light absorption ability can significantly impact the sensitivity and selectivity of the sensor. Thus, a comprehensive understanding of the underlying principles governing the GND-phosphate interaction could inform the design of phosphate ion sensors with the targeted performance. In line with this, the current study aims to investigate the influence of the strength of the interaction between graphene nanodots and phosphate ions on the nature of the absorption spectra of the complexes, using density functional theory (DFT) and TD-DFT calculations. The focus of this study will be on investigating the influence of carbon vacancies and metal (Al, Mg and Ca) dopant atoms on the adsorption capacity of GND towards phosphate ions. The choice of Al-, Mg-, and Ca-doped carbonous materials in theoretical simulations was motivated by their potential application in sensing [40,42,43,44]. Furthermore, the inclusion of diverse GND modifications allows for a comprehensive exploration of the factors affecting the sensing capabilities of GND-based systems. The results of this study can provide valuable insights into the optical properties of GND-phosphate complexes, which can help in designing and optimizing colorimetric sensing techniques for the detection of phosphates.

## 2. Methods

**Figure 1:**
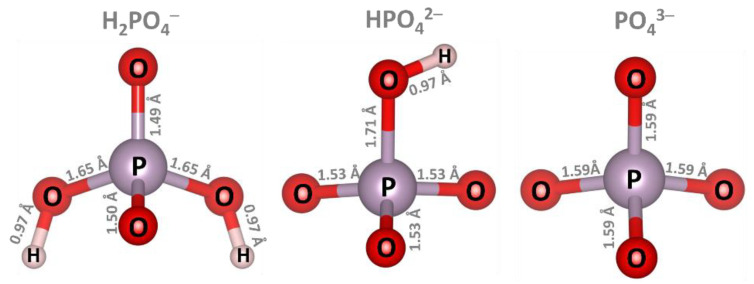
Optimized structures of the isolated phosphate ions, which were analyzed for key structural features, such as bond lengths.

DFT calculations were performed using the Gaussian 16 Rev. C.01 program package [45]. The neutral (closed shell) polycyclic aromatic hydrocarbon (PAH) molecule, C_96_H_24_, commonly known as circumcircumcoronene [46], was used as a representative sensing material model of graphene nanodots. Its selection as a core molecule for GND design is well-justified, given its suitability for investigating adsorption phenomena and exploring excited state properties of GND [47,48,49]. Therefore, C_96_H_24_ serves as a reasonable and appropriate choice for this study. Vacancy-containing and metal-doped GND structures (hereafter referred to as vac-GND, Ca-GND, Mg-GND and Al-GND, respectively) were created using the GaussView6 tool [50] through a simple removal of one carbon atom from GND or a replacement of the GND’s carbon atom by metal atoms (Ca, Mg, Al). The structures were then optimized using a geometry optimization calculation in the presence of water solvent. Adsorption of the phosphate ions on geometrically optimized GND surfaces in aqueous solution was modeled as the initial step causing the interaction between the sensing material and the target analyte in a real-life environment. H_2_PO_4_^−^, HPO_4_^2−^ and PO_4_^3−^ negatively charged ions (anions) were selected to simulate the phosphate ions (Figure 1). It is important to note that phosphate anions exhibit different protonation states depending on the pH of the solution in which they are present [51,52,53]. In acidic environments, the likelihood of phosphate ions existing in a protonated state is higher due to the relatively high concentration of protons (H^+^). This favors the complete protonation of the phosphate ion (H_3_PO_4_). As the pH becomes more alkaline, the gradual dissociation of protons from the phosphate molecule occurs, leading to partial or complete deprotonation and the formation of different species such as H_2_PO_4_^−^, HPO_4_^2−^ and PO_4_^3−^, depending on the number of protons released. However, it should be noted that in the current research, the specific investigation or consideration of the degree of deprotonation of phosphates under varying solution pH conditions was not conducted. The primary focus of the study was on performing DFT and TD-DFT calculations in the presence of water. In aqueous environments, the probability of phosphate ions being deprotonated and existing as dihydrogen phosphate (H_2_PO_4_^−^) or hydrogen phosphate (HPO4^2−^) species is increased. However, it is important to acknowledge that the presence of calcium cations (Ca^2+^) can significantly reduce the barrier for hydrogen phosphate deprotonation [52], suggesting the potential existence of phosphate anions (PO_4_^3−^) in real water samples as well. Considering these factors, the investigated reactions in this study demonstrate a high degree of likelihood, and the findings obtained hold valuable potential for the development of technologies aimed at monitoring phosphates in water. However, it is recommended that future investigations explore this aspect further to gain a deeper understanding of phosphate behavior under varying pH conditions and its impact on adsorption phenomena.

The adsorption energy of the phosphate ions on GND in the presence of water was calculated using the formula:

Δ*E*_ads_ = *E*_PI-GND_ − *E_P_*_I_ − *E*_GND_ (1)

where *E*_PI-GND_, *E_P_*_I_, and *E*_GND_ are the total energies of the phosphate-GND complex, isolated adsorbed phosphate ion, and isolated GND, respectively. The optimization was performed using the long-range-corrected CAM-B3LYP functional [54] (in combination with 6-31G(d) basis sets for all atoms [55]) until the default convergence criteria were reached.

The CAM-B3LYP functional was chosen for its good accuracy in predicting the electronic properties of graphene-based molecules [56,57,58,59]. Complemented by the widely used 6-31G(d) basis sets, it is the appropriate tool for computing small- to medium-sized molecules [60,61]. Moreover, the utilization of 6-31G(d) basis sets proved to be highly effective in offering a theoretically reliable description of the adsorption properties of composite materials when it comes to toxic substances, with their performance aligned with experimental verifications [62]. A self-consistent reaction field (SCRF) method, utilizing the polarizable continuum model (PCM), was used to take the water solvent effect into account [63]. The final optimized structures were checked for stability by calculating the vibrational frequencies.

TD-DFT calculations [64] were carried out on the optimized geometries of the GND systems and phosphate-GND complexes in the presence of water to predict their absorption spectra. The same level of theory as the ground-state DFT calculations was employed. The 100 excited states included in the calculation are sufficient to capture the main features of the absorption spectra. The calculated spectra correspond to the molar absorption coefficient (ε, L mol^−1^ cm^−1^) [65]. However, the relative intensity trends and spectral features are still valid and provide valuable insights into the absorption characteristics of the studied molecules. Oscillator strength analysis combined with frontier molecular orbital (FMO) analysis was performed to further understand the excited-state properties of the phosphate-GND complexes. Charge population analysis was performed using an atomic dipole-corrected Hirshfeld (ADCH) method [66].

## 3. Results and Discussion

**Figure 2:**
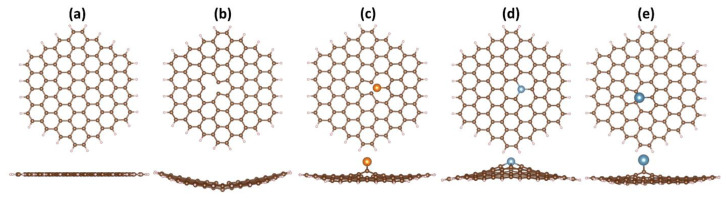
The optimized structures of various GND systems, including pristine GND (**a**), vac-GND (**b**), Mg-GND (**c**), Al-GND (**d**), and Ca-GND (**e**), are depicted in both top and side views. In these representations, carbon atoms are represented by brown-colored balls, hydrogen atoms by whitish-colored balls, and magnesium atoms by orange-colored balls. Additionally, aluminum atoms are depicted as small, blue-colored balls, while calcium atoms are represented by larger, blue-colored balls.

The optimized geometries of GND and its modified counterparts, including vac-GND, Ca-GND, Mg-GND, and Al-GND, were obtained by performing PCM/CAM-B3LYP/6-31G(d) calculations. The top and side views of the optimized structures are depicted in Figure 2. The pristine GND exhibits a regular hexagonal lattice with a flat structure, consistent with the PAH molecule (Figure 2a). However, upon the carbon vacancy’s formation and the introduction of metal atoms (Ca, Mg or Al), noticeable structural deviations become apparent. These structural deviations are manifested as a pronounced buckling or curvature in the initially flat GND, accompanied by local changes in the bonding environment. In the vacancy-containing GND structure, the carbon atoms neighboring the vacant site undergo rearrangement in an attempt to fill the vacancy, resulting in a pronounced deviation from the planar geometry and inducing significant buckling effects (Figure 2b). In turn, to accommodate the metal dopants, the carbon atoms of GND experience both upward and downward collective displacements (Figure 2c–e). Furthermore, a distortion of the GND plane is accompanied by the protrusion of the metal atoms from the surface (Figure 2c–e). The bond lengths of the calcium atom with the carbon atoms are 2.828, 2.780, and 2.571 Å. On the contrary, the magnesium atom forms two bonds with carbon atoms of equal length (2.282 Å) and one shorter bond (2.111 Å), while three equidistant Al-C bonds (1.835 Å) are observed when aluminum replaces the carbon atom in GND. The larger protrusion of metal atom observed in Ca-doped GND compared to Mg-GND and Al-GND complexes can be attributed to a larger ionic radius of Ca compared to Mg and Al [67].

The larger size of the Ca atom exerts greater strain on the neighboring C-C bonds, leading to more pronounced out-of-plane deformations in the GND structure. The Δ*z*_1_ and Δ*z*_2_ parameters are then calculated to quantify the degree of buckling and curvature (Table 1). Δ*z*_1_ represents the difference between the average *z*-coordinate and the absolute highest *z*-coordinate, while Δ*z*_2_ corresponds to the maximum deviation from the average. The pristine GND demonstrates a minimal buckling, as indicated by negligibly small Δ*z*_1_ and Δ*z*_2_ values. Meanwhile, these parameters demonstrate an increasing trend for modified GND structures, especially in the case of metal-doped GND. In the latter scenario, the degree of curvature correlates with the height at which the atom protrudes.

**Figure 3:**
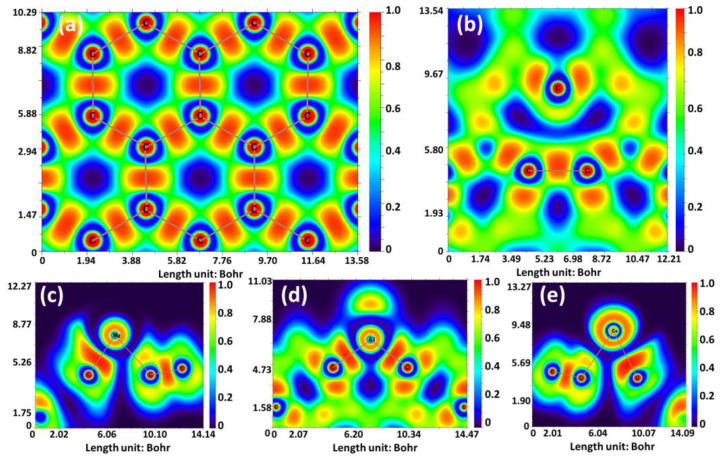
ELF contours for the GND (**a**), vac-GND (**b**), Mg-GND (**c**), Al-GND (**d**), and Ca-GND I (**e**)**.** ELF calculations were performed using the Multiwfn program [69].

To gain further insights into the bonding mechanism within the considered GND systems, electron localization function (ELF) [68] was also employed. These calculations were carried out to analyze the distribution of electrons and their localization in the examined GND systems, as depicted in Figure 3. The ELF contour in Figure 3 provides a visual representation of the degree of electron localization, with varying color scales. In the ELF contour, a value of 1 (red) indicates complete electron localization, while a value of 0.5 (green) represents the presence of electron-gas-like pairs. Conversely, a value of 0 (blue) signifies complete electron delocalization. Starting with pristine GND shown in Figure 3a, the ELF pattern demonstrates a uniform distribution of electron density, primarily concentrated between the C-C bonds. Upon introducing vacancies in the GND system, one can observe the emergence of extended blue regions in the vicinity of the carbon vacancy (Figure 3b). This observation suggests a significant delocalization of electron clouds in these areas. Finally, in the case of metal-doped GND systems (Figure 3c–e), a higher ELF value is observed in the region adjacent to the metal atoms. This observation indicates a propensity towards the formation of carbon-metal chemical bonds.

**Figure 4:**
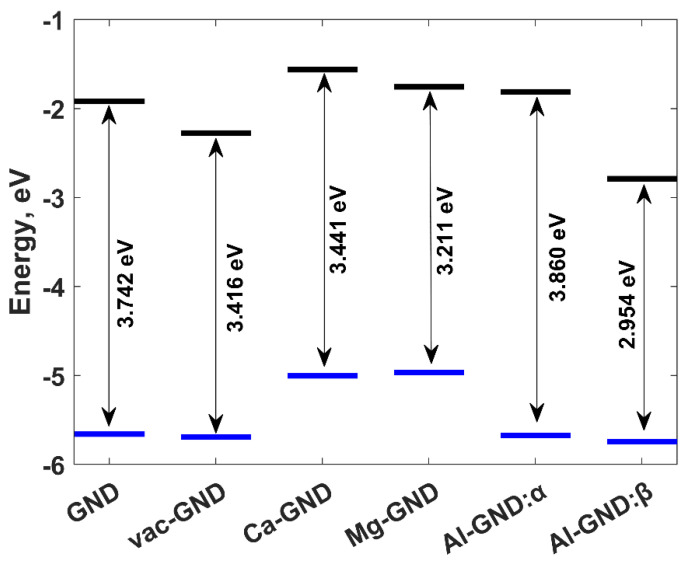
Molecular orbital diagram that summarizes the various systems under consideration. The energetic positions of the HOMO and LUMO levels are depicted by solid lines in blue and black, respectively. The HOMO-LUMO gaps are indicated by arrows.

The formation of vacancies and the substitution of metal atoms in the GND system exert significant influences on its electronic properties, namely the energy difference between the highest occupied molecular orbital (HOMO) and the lowest unoccupied molecular orbital (LUMO) (Figure 4). The HOMO-LUMO gap, which serves as a crucial indicator of adsorption efficiency [70], exhibited values of 3.742 eV for pristine GND, 3.416 eV for vacancy-containing GND, 3.441 eV for Ca-doped GND, and 3.211 eV for Mg-doped GND. These findings highlight the variations in the HOMO-LUMO gaps among different GND configurations, indicating their potential influence on the adsorption properties. The observed energy gap narrowing is mainly affected by the upward shift of the HOMO level, while the downward shift of LUMO acquires a less pronounced character. The narrowing of the observed energy gap is primarily influenced by the downward shift of the LUMO level in the case of the vacancy-containing GND (vac-GND). Conversely, in the Ca-GND and Al-GND systems, the upward shift of the HOMO level exhibits a more pronounced character, contributing to the narrowing of the energy gap. Interestingly, in contrast to the aforementioned structures where the HOMO and LUMO levels possess identical energies for both spin orientations, the introduction of aluminum dopants in GND leads to an energetic splitting of the HOMO and LUMO levels for spin-up (*α*) and spin-down (*β*) electrons. This phenomenon arises due to the presence of spin-dependent interactions and subsequent spin polarization within the Al-doped GND system. Notably, the partially filled *d*-shell of aluminum facilitates an exchange coupling between the localized aluminum spins and the itinerant carbon electron spins. Furthermore, upon immersion of the Al-doped GND molecule in water, the HOMO-LUMO gaps were observed to be 3.860 eV for the *α* mode and 2.954 eV for the *β* mode (Figure 4).

In the context of adsorption, the behavior of phosphate ions on the surfaces of the investigated systems is worth considering. Analysis of Table 1 reveals that the adsorbates have minimal impact on inducing out-of-plane deformations in the GND systems. Surprisingly, the structural integrity and shape of the GND remain almost intact following ion adsorption.

**Figure 5:**
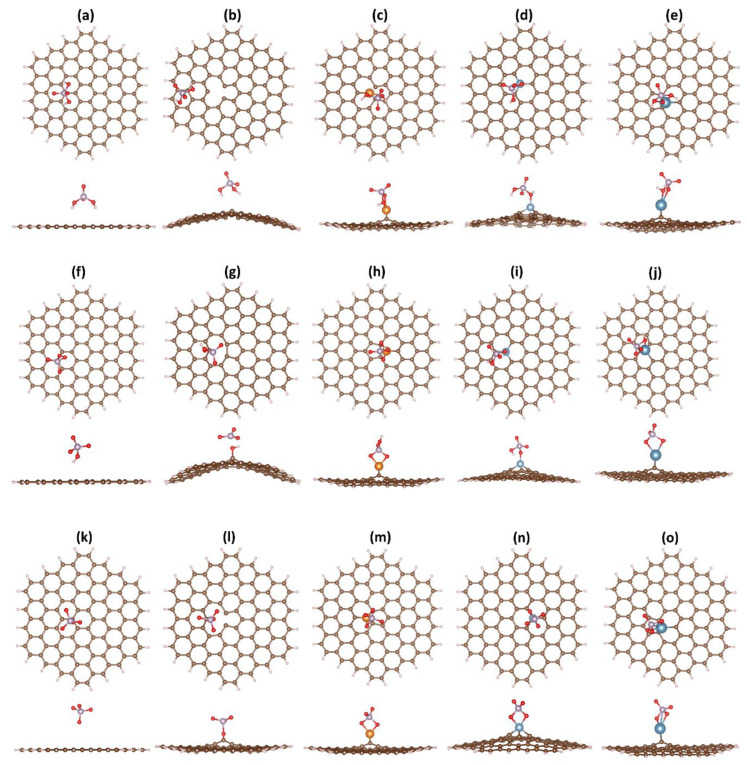
Optimized structures of various graphene nanodot (GND) types (pristine GND, vac-GND, Mg-GND, Al-GND, and Ca-GND) shown from top and side views, following adsorption of three phosphate ions: (**a**–**e**) H_2_PO_4_^−^, (**f**–**j**) HPO_4_^2−^, and (**k**–**o**) PO_4_^3−^. The color scheme employed in Figure 2 is utilized while incorporating the additional representation of oxygen and phosphorus atoms as red and violet balls, respectively.

For pristine GND (Figure 5a), the hydrogen atoms of the H_2_PO_4_^−^ molecule are directed towards the GND surface, while the phosphorus atom is positioned above a carbon atom. Interestingly, despite the initial placement of the H_2_PO_4_^−^ molecule directly above a missing carbon atom in vac-GND, it undergoes a shift towards the edge of the GND during the optimization process (Figure 5b). This shift suggests a weak interaction, potentially somewhat repulsive, between the H_2_PO_4_^−^ molecule and the carbon vacancy.

Examining metal-doped graphene nanodots, it is evident that the metal atom forms bonds with one or two oxygen atoms (Figure 5c–e). In this regard, the bond lengths of Ca-O and Mg-O (2.50 and 2.10 Å, respectively) significantly exceed the bond length of Al-O (1.88 Å). The adsorption configurations of HPO_4_^2−^ and PO_4_^3−^ ions on the nanodot surface (Figure 5f–o) closely resemble the adsorption configurations of the H_2_PO_4_^−^ molecule, with the exception that vac-GND forms stronger bonds with HPO_4_^2−^ and PO_4_^3−^ than with H_2_PO4^−^ (Figure 5g–i). Additionally, Figure 5n shows that the adsorption of the PO_4_^3−^ ion results in the formation of two Al-O bonds (1.86 and 1.93 Å), instead of one as observed in the H_2_PO4^−^@Al-GND and HPO_4_^2−^@Al-GND systems. An interesting observation is the significant reduction in the bond lengths of Ca-O and Mg-O with an increase in the negative charge of the ion: from 2.50 and 2.10 Å for the H_2_PO4^−^@Ca-GND and H_2_PO4^−^@Mg-GND systems to 2.29 and 1.95 Å for the PO_4_^3−^@Ca-GND and PO_4_^3−^@Mg-GND systems. The intermediate parameters for H_2_PO_4_^−^@Ca-GND and H_2_PO_4_^−^@Mg-GND are 2.30–2.43 and 2.01 Å, respectively.

**Figure 6:**
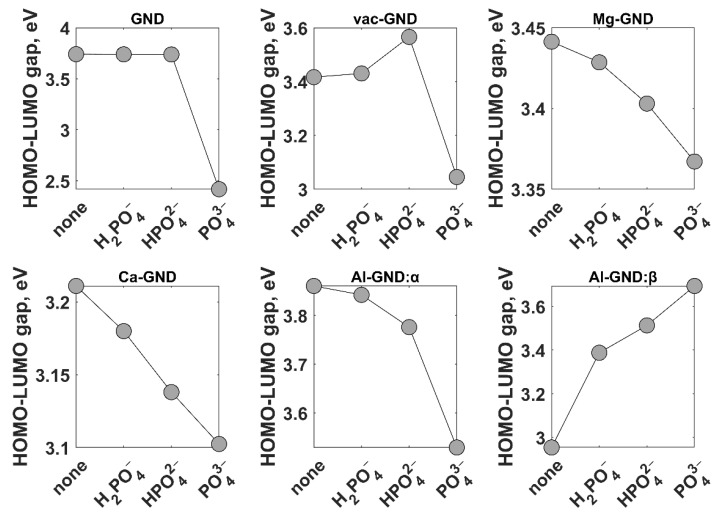
Effect of phosphate ion adsorption on HOMO-LUMO gap in various GND systems.

Moving beyond the structural characterization of phosphate ions on both pristine and modified GND surfaces, a comprehensive analysis of the adsorption energies and the magnitude of charge transfer between these ions and the surface becomes imperative. These factors assume a pivotal role in defining the sensing performance, making their evaluation vital. More specifically, the adsorption energy determines the strength of binding between the phosphate ion and the GND surface, consequently influencing the recovery time. A higher adsorption energy leads to a prolonged recovery process. Similarly, the degree of charge transfer between the adsorbed phosphate ions and the GND surface governs the sensor’s response. Hence, to achieve an optimal balance between rapid response and swift recovery, it is advantageous to have a substantial electron transfer coupled with a minimal adsorption energy [71]. DFT results presented in Table 2 demonstrate relatively low adsorption energies for phosphate ions on different structures of graphene nanodots, all falling below the commonly recognized lower chemisorption limit of 0.5 eV [72]. However, it is noteworthy that pure physisorption is observed only for phosphate ion adsorption on pristine GND and H_2_PO_4_^−^ adsorption on vac-GND. Physisorption arises from weak intermolecular forces, resulting in low adsorption energy and minimal electronic or chemical interactions. Conversely, chemical adsorption involves stronger chemical bonds (1–3 Å), leading to higher adsorption energy, substantial electronic and chemical interactions, and increased stability and specificity. Furthermore, when examining other GND surfaces, although the adsorption energies of phosphate ions remain below the 0.5 eV threshold, the equilibrium distance between the ions and the surface falls within the 1–3 Å range, indicating a significant interfacial electron transfer, as illustrated in Table 2. Thus, this case can be appropriately classified as weak chemisorption. Table 2 provides a comprehensive overview of the charge magnitudes observed on phosphate ions following their interaction with GND. The table highlights the deviations of the remaining charges on adsorbed phosphate ions from their initial charges (−1, −2, and −3, respectively) as individual isolated phosphate ions. These deviations serve as clear indications of electron redistribution within the GND-phosphate ion complexes, resulting in an electron transfer between the GND system and the adsorbed ions. Notably, as the charge values shift towards less negative charges in comparison to the ideal charge state, it signifies an electron transfer from the phosphate ion to the GND system. The careful analysis of these results also reveals several important trends. Firstly, the adsorption energies of all phosphate ions on modified GND surfaces were generally higher than on pristine GND, indicating enhanced adsorption affinity due to surface modifications. Secondly, among the modified GNDs, aluminum modification (Al-GND) exhibited the strongest adsorption energies for all three phosphate ions, suggesting its superior adsorption capability. Thirdly, there was a decreasing trend in adsorption energy with the size of the phosphate ion, with PO_4_^3−^ ions exhibiting the highest adsorption energies, followed by HPO_4_^2−^ ions, and H_2_PO_4_^−^ ions having the lowest adsorption energies. Finally, as we move from pristine GND to modified GND, the remaining charges on phosphate ions decrease, becoming less negative. Indeed, the observed trends indicate that phosphate ions undergo electron loss during the adsorption process. This also suggests that the modified GND surfaces, such as vac-GND, Ca-GND, Mg-GND, and Al-GND, which exhibit significant electron transfer with relatively low adsorption energies, may possess favorable characteristics for applications requiring both efficient electron transfer and easy desorption or recovery of the phosphate ions. The modification of the charge distribution caused by the adsorbed ions resulted in the changes in HOMO-LUMO energy gaps (Figure 6). Upon adsorption of H_2_PO_4_^−^ and HPO_4_^2−^, the energy gap of pristine GND experiences minor variations, suggesting a weak interaction between the ions and the graphene surfaces. However, with the adsorption of PO_4_^3−^ on GND, a more pronounced effect on the energy gap is observed. More specifically, the energy gap decreases further compared to the pristine GND case. In the case of vac-GND, the energy gap widens after the adsorption of H_2_PO_4_^−^ and HPO_4_^2−^ ions but undergoes a substantial reduction following the adsorption of more negatively charged PO_4_^3−^ ions. Interestingly, the trends within metal-doped systems reveal notable patterns. For Ca-GND, Mg-GND, and Al-GND (in alpha mode), the energy gaps decrease progressively from H_2_PO_4_^−^ to HPO_4_^2−^ to PO_4_^3−^ adsorption. This implies an increasing influence of the adsorbed ions on the electronic properties of these systems, leading to a more pronounced impact on the energy gap. It is worth highlighting the distinct behavior of Al-GND, where the energy gaps differ significantly between the alpha and beta modes after the adsorption of phosphate ions. This suggests the presence of spin-dependent effects and spin polarization induced by the combination of aluminum dopants and phosphate ions.

The energy gap of GND systems demonstrates a profound correlation with the size of the adsorbed phosphate ions on their surface, implying that the interaction between GND and phosphate ions can lead to significant modifications in their absorption spectra.

This holds great significance, particularly in the realm of optical sensor development for monitoring phosphate presence in water. Consequently, the following sections will discuss the results of TD-DFT investigations, shedding light on the diverse aspects of the impact of GND-phosphate ion interactions on the absorption spectra of GND. Through the elucidation of these findings, valuable contributions can be made towards advancing knowledge in the field of optical sensing of phosphates. In this regard, it is crucial to conduct an in-depth analysis of the intrinsic optical behavior exhibited by both pristine and modified GND prior to phosphate ion adsorption. This analysis will serve as a fundamental reference point for interpreting any subsequent alterations observed in the optical response after the adsorption of phosphate ions.

**Figure 7:**
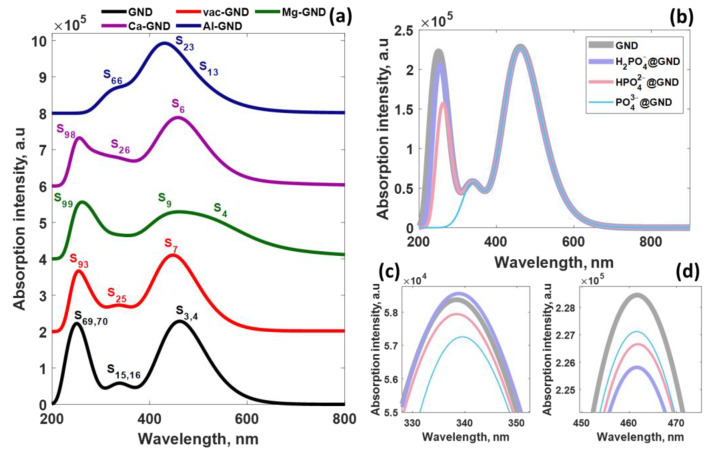
(**a**) UV–vis absorption spectra of the pristine and modified GND structures predicted using TD-DFT/PCM/CAM-B3LYP/6-31G* method before phosphate ion adsorption. (**b**) UV–vis spectra of pristine GND after phosphate ion adsorption, with zoomed spectral regions (**c**) 325–355 nm and (**d**) 450–475 nm.

The absorption spectrum of pristine GND is dominated by two spectral bands with peaks at 252 nm and 462 nm, but a weak absorption band at 343 nm is also distinguishable (Figure 7). Since the pristine GND is a highly symmetric molecule, the first two spectral features are assigned to doubly degenerate excited-state configurations (S_69,70_ ← S_0_, and S_3,4_ ← S_0_ with high oscillator strength), while the third one is related to S_15,16_ ← S_0_ transitions with a much lower oscillator strength of 0.48 (see also Table 3, which summarizes information on the five excited states with the largest oscillator strengths). It is important to note that the energy of the dominating excited states S_3,4_ is lower than the HOMO-LUMO gap, which can be related to the phenomenon of electron-hole attraction during excitation. Indeed, in the excited state, the electron and hole can experience attractive forces, which can reduce the overall energy of the excited state compared to the HOMO-LUMO gap. This electron-hole attraction is particularly relevant in cases where the electron and hole are spatially close to each other. This effect is precisely observed in the case of pristine GND [47]. The absorption spectrum of the pristine ground state (GND) undergoes significant alterations following modification (refer to Figure 7 and Table 3), primarily manifested by a notable reduction in the oscillator strength associated with the major electronic transitions. Although the shape of the absorption spectrum of vac-GND resembles, to some extent, that of the pristine GND, it is apparent that there is a variation in the contributions of the excited states to the overall absorption spectrum. More specifically, S_7_ and S_93_ are found to be most prominent in visible and UV regions, respectively. Moreover, it is worth noting that the primary peak in the long-wavelength region of the spectrum exhibits a distinct blue shift, moving towards shorter wavelengths. Certainly, this phenomenon is directly linked to a notable decrease in the degree of electron/hole delocalization (and hence the electron-hole attraction weakening) in the system when vacancy is introduced. Conversely, the UV band shows a noticeable redshift, characterized by a shift towards longer wavelengths.

A quick look at the evolution of the absorption spectra of metal-doped GND systems shows that the dopant-induced symmetry breaking leads to broadening of the main absorption bands of pristine GND and causes their shift, as can be clearly seen in the case of low-symmetry systems (Mg-GND and Ca-GND). Simultaneously, the Al-GND system demonstrates a prominent absorption band within the visible range, accompanied by a subtle shoulder in the shorter wavelength region of the spectrum. A comprehensive analysis of each spectrum reveals intriguing insights into the excited states of the Mg-GND, Ca-GND, and Al-GND systems. In the Mg-GND system, the most prominent and intense band spanning from 350 nm to 760 nm can be attributed to the S_3_, S_4_, and S_9_ excited states, respectively. Moving to the Ca-GND system, it exhibits relatively similar spectral characteristics to pristine GND and vac-GND, with significant contributions from the S_5_, S_6_, and S_7_ excited states.

The UV absorption baId in the Ca-GND system is primarily influenced by the S_98_ state. On the other hand, the absorption spectrum of Al-GND is dominated by a broad band centered at 434 nm, with the primary contributions originating from the S_23_ ← S_0_ and S_24_ ← S_0_ transitions. Additionally, a weak shoulder around 334 nm can be attributed to the S_66_ ← S_0_ transition. Notably, in the singlet excited state (S_23_), the promotion of electrons occurs from occupied molecular orbitals with a spin-down configuration to unoccupied molecular orbitals (also spin-down), resulting in a singlet state characterized by parallel spins. In contrast, the S_24_ state involves the promotion of electrons from both α and β occupied molecular orbitals to an unoccupied molecular orbital with a similar spin configuration. Furthermore, the transitions between molecular orbitals with spin-up configurations contribute significantly to the S_66_ state. A summary of the most probable electronic transitions involved in these excited states can be found in Table 3.

It becomes pertinent to inquire about the alterations occurring in the absorption spectra of GND systems when phosphate ions are introduced. The absorption spectrum of the GND exhibits notable modifications, mainly in the middle-ultraviolet region, following the phosphate ion adsorption, as illustrated in Figure 7b (see also Tables S1–S3, Supplementary Materials). However, the spectral characteristics in the near-ultraviolet and visible regions remain largely unchanged, with only marginal variations in band intensities, as depicted in Figure 7c,d. Upon closer examination of the absorption band within the middle-ultraviolet range, it becomes evident that an increase in the negative charge on the anion leads to a gradual reduction in band amplitude and a redshift of the peak. Ultimately, after the PO_4_^3−^ adsorption, the band completely disappears.

**Figure 8:**
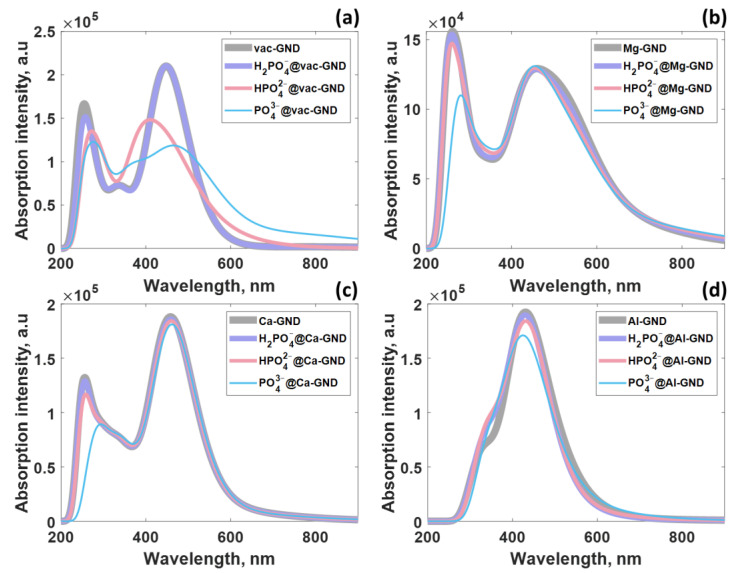
UV–vis spectra of modified GND after phosphate ion adsorption: (**a**) vac-GND, (**b**) Mg-GND, (**c**) Ca-GND, and (**d**) Al-GND.

In the context of this study, the successive interaction between vac-GND and each of the three ions under consideration leads to the emergence of three distinct absorption spectra (Figure 8a). These spectra exhibit a unique combination of excited states, as clearly demonstrated in Tables S1–S3 provided in the Supplementary Materials. This outcome forms a robust basis for effectively distinguishing and quantifying phosphate ions. When examining the absorption spectra of the Mg-GND and Ca-GND systems, a parallel trend to that observed in pristine GND becomes apparent. Specifically, the UV region displays notable sensitivity towards phosphates, as depicted in Figure 8b,c and further supported by the data in Tables S1–S3 found in the Supplementary Materials.

Turning our attention to the Al-GND system (Figure 8d), it is evident that this particular system exhibits lower sensitivity to the presence of phosphates compared to the others. This observation is indicated by a moderate negative charge effect on the anion, manifesting both in the visible part of the spectrum and its shorter wavelength segment. However, upon careful analysis of Tables S1–S3, it becomes evident that while the excited states involved in the formation of the primary absorption band in Al-GND remain largely unchanged after the adsorption of H_2_PO_4_^−^ and HPO_4_^2−^ ions, the adsorption of PO_4_^3−^ gives rise to a distinct set of excited states and a noticeable reduction in oscillator strengths for the primary electronic transitions. These characteristics, however, do not preclude the utilization of Al-GND for monitoring the presence of PO_4_^3−^.

The distinctive changes observed in the absorption spectra of GND, Mg-GND, and Ca-GND upon phosphate ion adsorption, particularly in the middle-ultraviolet region, offer potential for optical sensing of phosphates. The stable spectral characteristics in the near-ultraviolet and visible regions, with minimal intensity variations, provide a reference baseline for comparison. By focusing on the absorption band within the middle-ultraviolet range, it is possible to utilize the gradual reduction in band amplitude and the redshift of the peak as indicators of phosphate ion concentration. The disappearance/quenching of the absorption band entirely after PO_4_^3−^ adsorption serves as a clear signal of phosphate presence. These spectral modifications can be employed as optical signatures for the detection and quantification of phosphates, enabling the development of sensitive and selective optical sensing platforms. Considering the information given, GND, vac-GND, Ca-GND, Al-GND, and Mg-GND present themselves as viable options for constructing a sensing configuration that incorporates these elements into the sensor design. By harnessing the sensitivity of sensing elements’ UV–vis absorption to phosphate ions, along with employing suitable excitation sources, photodetection systems, and data analysis techniques such as multivariate analysis or pattern recognition algorithms, it becomes feasible to differentiate the three phosphate ions based on their unique spectral characteristics. In this manner, their distinctive spectral fingerprints can be utilized to discriminate between the phosphate ions effectively. Although the literature on this particular topic is still relatively scarce, it is evident that the phenomenon of pronounced property alterations in GND caused by the presence of PO_4_^3−^ ions extends beyond GND alone. Comparable behavior has been observed in other graphene-based materials, including negatively charged molybdate-mediated nitrogen-doped graphene quantum dots (Mo_7_O_24_^6−^ mediated N-GQDs) [73]. Remarkably, these materials have exhibited exceptional selectivity towards PO_4_^3−^, showcasing their superior adsorption capacity for PO_4_^3−^ in comparison to hydrogen phosphate and dihydrogen phosphate ions.

Table 4 serves to emphasize the distinct advantages and underscore the immense potential of GND-based optical sensor for PO_4_^3−^ ions detection. Although the existing literature primarily revolves around fluorescence-based techniques for detecting phosphate ions (in contrast to the absorption spectra discussed in this study), it is reasonable to deduce that performing a comparative analysis of parameters that characterize the alterations in optical signal amplitude of the quantum dots in the presence of phosphate ions would prove valuable. This analysis would provide insights into the effectiveness and sensitivity of sensors utilizing the investigated quantum dots, irrespective of the specific optical detection method employed. Table 4 provides compelling evidence that the interaction of a single phosphate ion with separate quantum dots, as explored in the current work, yields a notable relative change in the optical signal’s intensity, which is on par with the reported literature data for graphene quantum dot-based optical sensors specifically designed for PO_4_^3−^. This finding substantiates the effectiveness and performance of the proposed approach. Moreover, this method boasts a multitude of advantageous characteristics, such as the capability to detect individual phosphate ions, potential for high sensitivity, and the versatility and tunability offered by the graphene quantum dot system as well as its cost-effective fabrication process.

## 4. Conclusions

The research focus of this manuscript lies in exploring the interaction between graphene nanodots and phosphate ions for the purpose of optical sensing, demonstrating its significant impact on GND absorption spectra and identifying potential GND configurations for sensitive and selective phosphate detection. The introduction of vacancies and metal dopants in GND systems led to modifications in the absorption bands, broadening, and shifts in the wavelengths of the major electronic transitions. The adsorption of phosphate ions further modified the absorption spectra, particularly in the middle-ultraviolet region. The disappearance of the absorption band after phosphate ion adsorption served as a clear indication of phosphate presence. Based on the distinctive changes observed in the absorption spectra, GND, vac-GND, Ca-GND, Al-GND, and Mg-GND were identified as viable options for constructing optical sensing configurations. By utilizing the sensitivity of GND’s UV–vis absorption to phosphate ions, along with suitable excitation sources, photodetection systems, and data analysis techniques, it is feasible to differentiate and quantify phosphate ions based on their unique spectral characteristics. These findings contribute to the advancement of knowledge in the field of optical sensing and pave the way for the development of sensitive and selective optical sensing platforms for phosphate detection.

## Figures and Tables

**Table 1:** Parameters describing out-of-plane deformations of GND systems before and after phosphate ion adsorption.

Ion	GND		vac-GND		Ca-GND		Mg-GND		Al-GND	
	Δ*z*_1_, Å	Δ*z*_2_, Å	Δ*z*_1_, Å	Δ*z*_2_, Å	Δ*z*_1_, Å	Δ*z*_2_, Å	Δ*z*_1_, Å	Δ*z*_2_, Å	Δ*z*_1_, Å	Δ*z*_2_, Å
none	0.004	0.004	1.364	1.364	2.589	2.786	2.131	2.227	1.703	1.939
H_2_PO_4_^−^	0.389	0.390	1.556	1.556	1.724	2.747	1.436	2.216	1.126	2.056
HPO_4_^2−^	0.540	0.542	1.894	1.894	1.738	2.699	1.477	2.252	1.183	2.081
PO_4_^3−^	0.333	0.334	0.956	1.052	1.832	2.760	1.541	2.304	1.363	2.248

**Table 2:** Computed parameters (adsorption energy and remaining charge on ion) of pristine and modified GND after interaction with phosphate ions in water.

Structure	H_2_PO_4_^−^		HPO_4_^2−^		PO_4_^3−^	
	Δ*E*_ads_, eV	Charge, *e*^−^	Δ*E*_ads_, eV	Charge, *e*^−^	Δ*E*_ads_, eV	Charge, *e*^−^
GND	−0.0027	−1.0472	−0.0001	−2.0144	0.0011	−2.9916
vac-GND	−0.0039	−1.0481	−0.0016	−1.3113	−0.1614	−1.8274
Ca-GND	−0.0267	−0.6695	−0.0674	−1.4328	−0.1160	−2.3799
Mg-GND	−0.0441	−0.4845	−0.1147	−1.2604	−0.1669	−2.1696
Al-GND	−0.0709	−0.4487	−0.1389	−1.3032	−0.2001	−1.9408

**Table 3:** Properties of five dominant excited states in pristine and modified GND systems. The electronic transitions involving doubly degenerate excited states in pristine GND are included in square brackets.

*Structures*	*State*	*λ*, nm	*f*	*Assignment*
GND	S_3,4_	461.81	2.818	H-1→LUMO (21%), H-1→L + 1 (28%), HOMO→LUMO (28%), HOMO→L + 1 (21%) [H-1→LUMO (28%), H-1→L + 1 (21%), HOMO→LUMO (21%), HOMO→L + 1 (28%)]
	S_47,48_	271.93	0.648	H-9→L + 1 (12%), H-4→L + 2 (17%), H-3→L + 3 (17%), H-2→L + 2 (13%), HOMO→L + 8 (10%) [H-9→LUMO (12%), H-4→L + 3 (17%), H-3→L + 2 (17%), H-2→L + 3 (13%), H-1→L + 8 (10%)]
	S_69,70_	251.99	0.932	H-3→L + 5 (18%), H-3→L + 12 (12%) [H-4→L + 5 (18%), H-4→L + 12 (12%)]
	S_81,82_	244.70	0.814	H-3→L + 12 (27%) [H-4→L + 12 )27%]
	S_99,100_	229.12	0.784	H-18→LUMO (10%), H-3→L + 5 (10%), H-2→L + 10 (13%) [H-18→L + 1 (10%), H-4→L + 5 (10%), H-2→L + 11 (13%)]
vac-GND	S_7_	454.13	2.477	H-1→L + 2 (52%), HOMO→L + 1 (38%)
	S_8_	449.85	1.016	H-6→LUMO (11%), H-1→L + 1 (14%), HOMO→L + 2 (29%)
	S_9_	440.73	1.607	H-2→LUMO (26%), H-1→L + 1 (30%), HOMO→L + 2 (17%)
	S_93_	247.56	0.804	H-3→L + 11 (11%)
	S_99_	242.28	0.528	H-13→L + 1 (16%)
Ca-GND	S_5_	477.76	0.759	H-2→LUMO (24%), HOMO→L + 2 (10%), HOMO→L + 3 (35%)
	S_6_	461.45	1.835	H-2→L + 1 (31%), H-1→LUMO (36%)
	S_7_	457.67	1.566	H-1→L + 1 (28%), HOMO→L + 3 (24%)
	S_95_	249.54	0.322	H-17→L + 1 (2%), H-7→L + 10 (2%), H-6→L + 4 (4%), H-2→L + 15 (2%), H-2→L + 17 (2%), H-2→L + 18 (3%), H-1→L + 14 (2%), HOMO→L + 17 (3%), HOMO→L + 19 (9%)
	S_98_	247.30	0.366	H-15→L + 1 (3%), H-12→LUMO (3%), H-12→L + 3 (2%), H-10→L + 3 (3%), H-8→L + 3 (2%), H-6→L + 4 (6%), H-5→L + 6 (4%), H-5→L + 7 (3%), H-4→L + 8 (4%), H-3→L + 5 (2%), H-3→L + 6 (3%), H-3→L + 8 (2%), H-3→L + 9 (9%)
Mg-GND	S_3_	543.28	0.930	H-2→L + 1 (11%), H-1→LUMO (68%), HOMO→L + 1 (12%)
	S_4_	525.78	1.202	H-1→L + 1 (80%)
	S_9_	436.51	0.909	H-3→LUMO (31%), H-2→L + 1 (40%)
	S_92_	249.74	0.524	H-3→L + 12 (12%), H-1→L + 13 (10%)
	S_99_	245.58	0.626	HOMO→L + 22 (14%)
Al-GND	S_13_	507.01	0.694	H-2(A)→L + 1(A) (14%), H-1(A)→L + 1(A) (16%), HOMO(A)→LUMO(A) (20%), H-2(B)→LUMO(B) (19%)
	S_21_	438.10	0.299	H-2(A)→L + 1(A) (19%)
	S_23_	434.49	1.168	H-9(B)→LUMO(B) (10%), H-1(B)→L + 1(B) (12%), HOMO(B)→L + 2(B) (29%)
	S_24_	433.61	1.058	H-2(A)→LUMO(A) (13%), HOMO(B)→L + 1(B) (16%)
	S_25_	429.48	0.663	H-1(A)→L + 2(A) (14%), H-5(B)→LUMO(B) (24%)

**Table 4:** Comparison of graphene quantum dot-based optical sensors for phosphate ions. The following relationship, Δ*F*/*F*_0_ (%) ((*F* − *F*_0_)/*F*_0_ (%)), was employed to calculate the change in optical signal intensity. *F*_0_ (*F*) is the intensity of the optical signal before (after) phosphate ion adsorption.

Structure	Ref.	Sensor Concept	Limit of Detection	[(*F* − *F*_0_)/*F*_0_] × 100%
CVD graphene	[74]	Ion-sensitive field effect transistor with graphene/ionophore hybrid membrane for PO_4_^3−^ detection	0.1 mg/L	-
Mo_7_O_24_^6−^-mediated N-GQDs	[73]	A fluorescence turn-on probe for phosphate (PO_4_^3−^) ion detection	50 nM	30 at [1 μM)]
Single-layered s-GQDs-Al^3+^ system	[40]	Photoluminescence probe for highly selective PO_4_^3−^ detection	0.1 μM	13 at [7.5 μM)]
s-GQDs-Dy^3+^ complex	[75]	Off–on fluorescent strategy detection of PO4^3−^	0.1 μM	28 [at 0.2 μM]
Boron-doped GQDs with Fe^3+^ (B-GQDs)	[76]	Indirect fluorescent detection of PO4^3−^ (turn-off-on model)	340 nM	7 [at 3 μM]
Carbon dots (CDs)-Ce^3+^ ions ensembles	[77]	Triple-channel fluorescent sensor array for the identification of various phosphate anions (PO_4_^3−^)	10 μM	27 [at 10 μM]
Carbon dots (CDs)-Fe^3+^ ions ensembles	[77]	Triple-channel fluorescent sensor array for the identification of phosphate anions (PO_4_^3−^)	10 μM	6 [at 10 μM]
Nozzle-jet-printed silver/reduced graphene oxide (Ag/rGO)	[78]	Field-effect transistor phosphate ion (PO_4_^3−^) sensor	0.20 μM	-
Graphene quantum dots combined with Europium ions	[41]	Off–on photoluminescent probes for phosphate (PO_4_^3−^) sensing	0.1 μM	10 [at 0.5 μm]
Laser scribed reduced graphene oxide (LSGO)	[79]	Electrochemical PO_4_^3−^ sensing based on molybdenum blue method	0.0004 mM	-
Graphene quantum dots-Eu^3+^	[80]	Luminescent phosphate (PO_4_^3−^) sensor	100 nM	53 [at 1 μm]
vac-GND	this work	UV–vis spectroscopic method for detection of PO_4_^3−^	-	26
Mg-GND	this work	UV–vis spectroscopic method for detection of PO_4_^3−^	-	21
Al-GND	this work	UV–vis spectroscopic method for detection of PO_4_^3−^	-	11
Ca-GND	this work	UV–vis spectroscopic method for detection of PO_4_^3−^	-	33

## Data Availability

The data that support the findings of this study are available within the article.

## Supplementary Materials

The following supporting information can be downloaded at: https://www.mdpi.com/article/10.3390/s23125631/s1, Table S1: Properties of five dominant excited states in pristine and modified GND systems after H_2_PO_4_^−^ adsorption. The electronic transitions involving in doubly degenerate excited states in pristine GND are included in square brackets; Table S2: Properties of five dominant excited states in pristine and modified GND systems after HPO_4_^2−^ adsorption. The electronic transitions involving in doubly degenerate excited states in pristine GND are included in square brackets; Table S3: Properties of five dominant excited states in pristine and modified GND systems after PO_4_^3−^ adsorption. The electronic transitions involving in doubly degenerate excited states in pristine GND are included in square brackets.
