# Supplementary material for: A DFT Study of Phosphate Ion Adsorption on Graphene Nanodots: Implications for Sensing
Source: Sensors (Basel). 2023 Jun 16;23(12):5631. doi: 10.3390/s23125631 (PMC10303874; doi:10.3390/s23125631)
Supplement: Supplementary file 1 [file sensors-23-05631-s001.zip › sensors-2449613-supplementary.pdf]

Supplementary Materials

A DFT Study of Phosphate Ion Adsorption on Graphene Nanodots: Implications for Sensing

Ivan Shtepliuk

Semiconductor Materials Division, Department of Physics, Chemistry and Biology-IFM, Linköping University, S-58183 Linköping, Sweden; ivan.shtepliuk@liu.se

Table S1. Properties of five dominant excited states in pristine and modified GND systems after $H_2PO_4^-$adsorption. The electronic transitions involving in doubly degenerate excited states in pristine GND are included in square brackets.

Structures	***State***	***λ, nm***	***f***	***Assignment***
$H_2PO_4^-$@GND	$S_{3,4}$	461.76<br>[461.74]	2.789<br>[2.780]	H-1→LUMO (47%), HOMO→L+1 (47%)<br>[H-1→L+1 (48%), HOMO→LUMO (47%)]
	$S_{47,48}$	272.16<br>[272.06]	0.613<br>[0.617]	H-9→L+1 (12%), H-4→L+2 (23%), H-3→L+3 (21%), H-2→L+3 (16%), HOMO→L+8 (10%)<br>[H-9→LUMO (12%), H-4→L+3 (22%), H-3→L+2 (24%), H-2→L+2 (13%)]
	$S_{51,52}$	268.07<br>[268.05]	0.478<br>[0.462]	H-5→L+3 (10%), H-2→L+2 (37%), HOMO→L+9 (16%) [H-5→L+2 (10%), H-2→L+3 (35%), H-1→L+9 (15%)]
	$S_{71,72}$	252.33<br>[255.22]	0.850<br>[0.696]	H-3→L+5 (15%), H-3→L+12 (12%)<br>[H-4→L+5 (17%), H-4→L+12 (10%)]→
	$S_{84,85}$	244.76<br>[244.69]	0.666<br>[0.685]	H-3→L+12 (27%), H-1→L+13 (18%)<br>[H-11→LUMO (20%), H-4→L+12 (23%), H-1→L+14 (10%)]
$H_2PO_4^-$@ vac-GND	S_7	454.35	2.457	H-1→L+2 (50%), HOMO→L+1 (39%)
	S_8	448.76	1.373	H-1→L+1 (20%), HOMO→L+2 (34%)
	S_9	439.97	1.259	H-8→LUMO (10%), H-2→LUMO (29%), H-1→L+1 (24%), HOMO→L+2 (11%)
	S_{26}	338.81	0.358	H-6→L+1 (10%), H-3→L+4 (11%), HOMO→L+8 (15%)
	S_{97}	247.47	0.738	H-3→L+11 (10%)
$H_2PO_4^-$@ Ca-GND	S_5	479.46	0.705	H-2→LUMO (24%), HOMO→L+3 (37%)
	S_6	462.54	1.799	H-2→L+1 (29%), H-1→LUMO (35%)
	S_7	458.87	1.518	H-2→L+1 (10%), H-1→L+1 (27%), HOMO→L+3 (23%)
	S_{95}	249.78	0.370	H-15→LUMO (2%), H-6→L+4 (3%), H-3→L+8 (4%), H-3→L+11 (4%), H-2→L+12 (2%), H-2→L+15 (3%), H-2→L+17 (4%), H-1→L+14 (3%), H-1→L+22 (2%), HOMO→L+16 (3%), HOMO→L+19 (8%)
	S_{97}	248.21	0.275	H-14→LUMO (3%), H-13→L+1 (2%), H-12→L+2 (2%), H-10→L+4 (2%), H-9→L+3 (4%), H-7→L+2 (3%), H-6→L+2 (2%), H-6→L+4 (4%), H-3→L+6 (3%), H-2→L+14 (3%), H-1→L+17 (5%),

				HOMO→L+19 (3%), HOMO→L+21 (3%), HOMO→L+22 (4%)
$H_2PO_4^-$@ Mg-GND	S_3	547.92	0.840	H-2→L+1(12%),H-1→LUMO(69%),HOMO→L+1(10%)
	S_4	528.33	1.126	H-1→L+1 (81%)
	S_7	442.48	0.520	H-2→LUMO (30%), HOMO→L+4 (35%)
	S_9	437.99	0.972	H-3→LUMO (28%), H-2→L+1 (41%)
	S_{94}	246.92	0.686	H-3→L+5 (10%), HOMO→L+20 (14%)
$H_2PO_4^-$@ Al-GND	S_{18}	445.44	0.707	H-2(A)→L+1(A) (30%), H-3(B)→LUMO(B) (16%)
	S_{19}	441.06	0.608	H-1(A)→L+1(A) (10%), H-2(B)→L+1(B) (18%), H-1(B)→L+2(B) (12%)
	S_{21}	438.33	0.995	HOMO(A)→L+3(A)(23%),HOMO(B)→L+2(B)(17%)
	S_{23}	430.60	0.914	H-2(A)→LUMO(A)(14%),HOMO(B)→L+1(B) (12%)
	S_{29}	401.95	0.579	H-7(B)→LUMO(B) (21%),HOMO(B)→L+4(B)(18%)

Table S2. Properties of five dominant excited states in pristine and modified GND systems after HPO_4^{2-}adsorption. The electronic transitions involving in doubly degenerate excited states in pristine GND are included in square brackets.

Structures	***State***	***λ, nm***	***f***	***Assignment***
HPO_4^{2-}@GND	$S_{3,4}$	462.02 [461.93]	2.781 [2.772]	H-1→LUMO (47%), HOMO→L+1 (47%) [H-1→L+1 (47%), HOMO→LUMO (47%)]
	$S_{22,23}$	342.51 [342.43]	0.419 [0.474]	H-4→L+2 (16%), HOMO→L+6 (24%), HOMO→L+9 (15%) [H-4→L+3 (17%), H-1→L+6 (15%), H-1→L+9 (17%), HOMO→L+7 (13%)]
	$S_{66,67}$	271.90 [271.85]	0.642 [0.648]	H-15→L+1 (10%), H-7→L+2 (10%), H-6→L+3 (17%) [H-15→LUMO (11%), H-7→L+3 (10%), H-6→L+2 (18%)]
	$S_{72,73}$	267.80 [267.71]	0.414 [0.416]	H-4→L+2 (34%), HOMO→L+9 (13%) H-4→L+3 (32%), H-1→L+9 (13%)
	$S_{97,98}$	252.08 [252.02]	0.86 [0.900]	H-17→LUMO (5%), H-16→L+1 (5%), H-15→LUMO (3%), H-9→L+1 (3%), H-8→L+3 (4%), H-7→L+5 (4%), H-7→L+12 (3%), H-6→L+5 (6%), H-6→L+12 (4%), H-5→L+5 (8%), H-5→L+12 (5%), H-4→L+2 (3%), H-4→L+10 (6%), H-1→L+14 (3%), H-1→L+20 (2%), HOMO→L+13 (3%), HOMO→L+18 (2%), HOMO→L+19 (2%) [H-6→L+5 (12%)]
HPO_4^{2-}@ vac-GND	S_6	472.84	1.121	H-1→L+1 (72%)
	S_{11}	424.38	0.481	H-4→L+1 (18%), H-2→L+1 (34%), HOMO→L+3 (13%)
	S_{12}	402.29	0.967	H-4→L+1 (24%), H-3→L+2 (21%), H-2→L+1 (16%)
	S_{15}	388.59	0.524	H-4→LUMO (18%), H-2→LUMO (11%), H-1→L+4 (29%)
	S_{19}	368.68	0.616	H-2→L+2 (24%), H-1→L+4 (13%), HOMO→L+3 (12%)
HPO_4^{2-}@ Ca-GND	S_5	483.31	0.589	H-2→LUMO (23%), HOMO→L+3 (39%)
	S_6	464.42	1.718	H-2→L+1 (24%), H-1→LUMO (32%), H-1→L+1 (12%)

	S_7	461.19	1.471	H-2→L+1 (14%), H-1→LUMO (11%), H-1→L+1 (23%), HOMO→L+3 (20%)
	S_8	450.19	0.431	H-3→LUMO (23%), HOMO→L+4 (23%)
	S_{96}	249.86	0.516	H-15→L+1 (3%), H-14→L+1 (2%), H-8→L+9 (2%), H-3→L+8 (7%), H-2→L+12 (3%), H-2→L+16 (4%), HOMO→L+18 (9%)
HPO_4^{2-}@ Mg-GND	S_3	554.86	0.748	H-2→L+1 (12%), H-1→LUMO (70%)
	S_4	533.01	1.021	H-1→L+1 (81%)
	S_8	444.18	0.760	H-2→LUMO (47%), HOMO→L+4 (12%)
	S_9	441.55	1.139	H-3→LUMO (19%), H-2→L+1 (47%)
	S_{95}	248.01	0.534	HOMO→L+19 (15%)
HPO_4^{2-}@ Al-GND	S_{18}	451.04	0.454	H-2(A)→LUMO(A) (17%)
	S_{19}	444.77	0.689	H-1(A)→LUMO(A) (10%), H-2(B)→L+1(B) (22%)
	S_{20}	444.19	0.974	HOMO(A)→L+3(A) (29%), HOMO(B)→L+2(B) (18%)
	S_{22}	433.08	0.732	H-2(A)→L+1(A) (10%), HOMO(B)→L+1(B) (11%)
	S_{29}	400.45	0.553	H-2(B)→L+1(B) (15%), HOMO(B)→L+4(B) (21%)

Table S3. Properties of five dominant excited states in pristine and modified GND systems after PO_4^{3-}adsorption. The electronic transitions involving in doubly degenerate excited states in pristine GND are included in square brackets.

Structures	***State***	***λ, nm***	***f***	***Assignment***
PO_4^{3-}@GND	$S_{18,19}$	461.73 [461.69]	2.801 [2.799]	H-4→L+1 (40%), H-3→LUMO (39%) [H-4→LUMO (40%), H-3→L+1 (40%)]
	$S_{64,65}$	342.17 [342.16]	0.486 [0.485]	H-10→L+2 (24%), H-4→L+7 (15%), H-3→L+6 (16%), H-3→L+9 (14%) [H-10→L+3 (24%), H-4→L+6 (17%), H-4→L+9 (14%), H-3→L+7 (15%)]
	$S_{70,71}$	333.26 [333.23]	0.198 [0.198]	H-14→LUMO (23%), H-11→L+4 (19%), H-4→L+8 (17%) [H-14→L+1 (23%), H-12→L+4 (19%), H-3→L+8 (17%)]
PO_4^{3-}@ vac-GND	S_4	529.07	0.992	H-1→LUMO (82%)
	S_8	460.51	1.016	H-2→LUMO (55%), H-1→L+1 (10%), HOMO→L+2 (19%)
	S_9	453.50	0.399	H-3→LUMO (21%), H-2→L+1 (37%), H-1→L+2 (10%), HOMO→L+4 (14%)
	S_{20}	368.33	0.467	H-4→L+1 (13%), HOMO→L+6 (13%), HOMO→L+8 (35%)
	S_{58}	285.29	0.406	H-10→L+1 (18%), H-2→L+5 (15%)
PO_4^{3-}@ Ca-GND	S_5	488.72	0.483	H-2→LUMO (23%), HOMO→L+3 (40%)
	S_6	467.68	1.585	H-2→LUMO (11%), H-2→L+1 (17%), H-1→LUMO (27%), H-1→L+1 (16%)
	S_7	465.44	1.224	H-5→LUMO (13%), H-2→L+1 (19%), H-1→LUMO (12%), H-1→L+1 (14%), HOMO→L+3 (17%)
	S_8	454.92	0.761	H-5→LUMO (22%), H-2→LUMO (10%), H-1→L+1 (16%), HOMO→L+4 (18%)
	S_{22}	369.28	0.304	HOMO→L+5 (54%)
PO_4^{3-}@ Mg-GND	S_3	560.60	0.669	H-2→L+1 (13%), H-1→LUMO (71%)
	S_4	536.91	0.933	H-1→L+1 (81%)
	S_8	447.80	0.951	H-2→LUMO (54%)
	S_9	444.57	1.221	H-3→LUMO (13%), H-2→L+1 (48%), HOMO→L+2 (12%)
	S_{74}	271.04	0.413	H-3→L+7 (17%)

PO_4^{3-}@ Al-GND	S_{23}	451.47	0.503	HOMO(A)→L+3(A) (12%), H-2(B)→L+2(B) (16%)
	S_{26}	442.23	0.448	HOMO(B)→L+2(B) (11%)
	S_{27}	437.81	0.385	H-2(A)→L+1(A) (10%)
	S_{29}	424.66	0.523	H-3(A)→LUMO(A) (11%)
	S_{32}	407.92	0.649	H-3(B)→L+1(B) (19%)
